# Evaluation of Shear Wave Velocity and Human Bone Morphogenetic Protein-7 for the Diagnosis of Diabetic Kidney Disease

**DOI:** 10.1371/journal.pone.0119713

**Published:** 2015-03-19

**Authors:** Ning Yu, Yue-yue Zhang, Xiao-yan Niu, Yan Xu, Rui-xia Ma, Wei Zhang, Xiu-bo Jiang

**Affiliations:** 1 Department of Ultrasound, The Affiliated Hospital of Qingdao University, Qingdao, Shandong, China; 2 Department of Nephrology, Shanghai Jiaotong University Affiliated First People’s Hospital, Shanghai, China; 3 Department of Nephrology, The Affiliated Hospital of Qingdao University, Qingdao, Shandong, China; 4 Department of Public Health Epidemiology of the Medical College of Qingdao University, Qingdao, Shandong, China; Charles P. Darby Children's Research Institute, 173 Ashley Avenue, Charleston, SC 29425, USA, UNITED STATES

## Abstract

**Purpose:**

The aim of this study was to determine the diagnostic values of kidney shear wave velocity (SWV) and bone morphogenetic protein-7 (BMP-7), and their correlation in the diagnosis of early diabetic kidney disease.

**Methods:**

A total of 150 patients with type 2 diabetes mellitus were divided into three equal groups based on the urinary albumin-creatinine ratio (ACR): normal albuminuria (normo- group, ACR < 30 mg/g creatinine, n = 50), microalbuminuria (micro- group, 30 ≤ ACR < 300 mg/g creatinine, n = 50), and macroalbuminuria (macro- group, ACR ≥ 300 mg/g creatinine and estimated glomerular filtration rate (eGFR) ≥30 ml/min/1.73 m^2^, n = 50). Fifty healthy volunteers were recruited to serve as controls (control group). The levels of serum BMP-7 were detected, and virtual touch tissue quantification was used to detect the renal SWV value in all study subjects. Correlations between groups as well as SWV and BMP-7 were analyzed.

**Results:**

Serum BMP-7 and SWV were significantly and progressively decreased and increased, respectively, during the development of renal disease, from the normo- to the micro- and to the macro- groups (all *P* < 0.01 between each other for BMP-7 and SWV). Moreover, no significant differences between the normo- and control groups were observed for either BMP-7 or SWV (both *P* > 0.05). In addition, a significant correlation was found between SWV and BMP-7, with a coefficient of -0.569 (*P* < 0.05).

**Conclusion:**

The determination of SWV together with serum BMP-7 may play an important role in the diagnosis of diabetic kidney disease.

## Introduction

Diabetic kidney disease (DKD), which is caused by glomerulosclerosis and renal interstitial fibrosis, is one of the most severe complications of type 2 diabetes mellitus (T2DM). As it usually has a poor prognosis, early diagnosis is crucial for the treatment of DKD with T2DM. It has been well established that renal fibrosis, as a precursor to end-stage renal disease, plays an essential role in the development of chronic kidney disease. Fibrosis causes the kidney to stiffen. To detect the extent of fibrosis or stiffness in renal tissue, virtual touch tissue quantification (VTQ) has been widely recommended, which is an ultrasound imaging technique that detects the elasticity/stiffness of tissues or organs [1–4]. Ultrasound transmits pulses automatically, inducing the target tissue to produce shear waves. The shear waves can be induced by a high-frequency transducer, collected, and then analyzed for the elasticity of the target tissue to quantify the extent of tissue fibrosis[5]. The technology has the following benefits: it is noninvasive and without ionizing radiation for deep tissues or organs, is inexpensive to use, produces consistently stable results with minimal error, and presents numerical data results[6]. There are many studies that have explored VTQ measurements as a means of evaluating renal tissue stiffness, including several studies on the kidneys. For example, Lee et al. [7], Holdsworth et al.[8], and Sohn et al.[9] measured the acoustic radiation force impulse velocities of normal kidneys in healthy adults. Furthermore, there have been several trials using shear waves in adult kidneys to evaluate renal masses[2], to assess renal allograft fibrosis[10], and to detect chronic kidney diseases[3].

Bone morphogenetic protein-7 (BMP-7), one of the endogenous hormones in the kidney, is a major inhibiting factor of the profibrotic transforming growth factor beta (TGF-β) and has a strong antifibrotic activity[11,12]. In addition, losses of tubular BMP-7 or serum BMP-7 have been observed in progressive diabetic nephropathy[13] or in direct correlation with a loss of viable renal mass[14], indicating that BMP-7 plays an important role in the pathogenesis of DKD. Michos et al. [15] have demonstrated that the endogenous expression of BMP-7 decreases as DKD progresses, thus promoting renal fibrosis in a mouse model. By giving exogenous recombinant BMP-7 to DKD rats, researchers have found reduced renal fibrosis, podocyte loss, kidney injury, oxidative stress, extracellular matrix accumulation, urinary protein, and glomerular filtration rate[16,17], suggesting that exogenous administration of BMP-7 may inhibit glomerulosclerosis and interstitial fibrosis, thereby improving the renal function in DKD.

We have previously studied VTQ to determine renal injury in T2DM and demonstrated that the shear wave velocity (SWV) increases as renal injury progresses[3]. However, it has been suggested that SWV alone may not be possible to accurately assess kidney fibrosis due to renal blood flow interference[18]. In addition, biochemical markers of DKD may require an extensive time lapse during which successful interventions could be tested and applied. Therefore, the combined examination of SWV and BMP-7 may have an additional diagnostic significance. The purpose of this study was to detect renal SWV and serum BMP-7 in different stages of DKD and to study the feasibility of these two parameters in the diagnosis of DKD renal fibrosis.

## Materials and Methods

### Study participants

This was a single-center study that prospectively enrolled 175 T2DM patients from the Endocrinology and Nephrology Departments of the Affiliated Hospital of Qingdao University from January 2012 to January 2013. This study was approved by the Human Research Ethics Committee of our hospital, and written informed consent was obtained from all the subjects involved in this study.

The inclusion criteria for patient enrolment in this study were in-hospital patients with T2DM, diagnosed based on the Standards of Medical Care in Diabetes–2010[19], with or without kidney disease. DKD was diagnosed based on the following K/DOQI guidelines and clinical practice recommendations for diabetes and chronic kidney disease[20]: (1) macroalbuminuria is present; or (2) microalbuminuria is present in the presence of diabetic retinopathy. Other causes of chronic kidney disease were considered in the presence of any of the following circumstances: (1) absence of diabetic retinopathy; (2) low or rapidly decreasing glomerular filtration rate (GFR); (3) rapidly increasing proteinuria or nephrotic syndrome; (4) refractory hypertension; (5) presence of active urinary sediment; (6) signs or symptoms of other systemic diseases; (7) >30% reduction in the GFR within 2–3 months after initiation of treatment with an angiotensin-converting enzyme inhibitor or angiotensin receptor blocker. The exclusion criteria were as follows: patients who had suffered from any nondiabetic renal diseases, had a systemic disease/condition that could influence the albumin-creatinine ratio (ACR) as assessed by medical history and medical examination, taking any medications that could affect renal function, or did not provide consent for the study. However, T2DM patients with existing co-moribidities such as peripheral vascular disease, coronary artery disease, liver disease etc. that may interfere with the analysis results were included in this study.

The DKD patients were divided into the following three groups according to the urinary ACR: normoalbuminuria (normo-group), ACR < 30 mg/g creatinine, including 29 male and 21 female patients (mean age ± standard deviation (SD), 48.65 ± 16.83 years old, range: 33–62 years old); microalbuminuria (micro- group), 30 ≤ ACR < 300 mg/g creatinine, including 25 male and 25 female patients (mean age ± SD, 50.42 ± 16.52 years old, range: 35–64 years old); macroalbuminuria (macro- group), ACR ≥ 300 mg/g creatinine, including 27 male and 23 female patients (mean age ± SD, 50.24 ± 15.72 years old, range: 38–62 years old). The control group consisted of 50 age-matched healthy volunteers (27 males and 23 females with a mean age ± SD of 52.54 ± 13.41 years old, range: 37–63 years old), who visited the medical centers of the Affiliated Hospital of Qingdao University for an annual health checkup, were randomly selected based on the normal clinical characteristics of blood pressure, blood glucose, blood lipids, renal function, and renal ultrasound. The estimated glomerular filtration rates (eGFRs) varied among the groups (Table 1); however, all the study subjects had eGFR values ≥ 30 ml/min/1.73 m^2^.

**Table 1:** Clinical characteristic of the study groups.

	Control group	Normo- group	Micro- group	Macro- group	*P*
Gender (male/female)	27/23	29/21	25/25	27/23	0.90
Age (years)	49.74 ± 7.89	48.95 ± 7.31	49.19 ± 6.89	50.32 ± 7.06	0.79
BMI (kg/m^2^)	25.01 ± 1.55	25.39 ± 1.34	25.38 ± 1.41	24.78 ± 1.24	0.07
eGFR (ml/min·1.73 m^2^)	136.42 ± 14.98	145.39 ± 26.53	93.54 ± 36.48	53.54 ± 15.6	< 0.01
fasting blood glucose (mmol/l)	5.53 ± 0.32	5.62 ± 0.32	5.58 ± 0.40	5.65 ± 0.30	0.30
HbA_1_C (%)	5.32 ± 0.69	5.20 ± 0.75	5.45 ± 0.69	5.53 ± 0.57	0.06
LDL (mmol/l)	2.36 ± 0.66	2.56 ± 0.58	2.59 ± 0.85	2.66 ± 0.66	0.57
HDL (mmol/l)	0.98 ± 0.38	1.13 ± 0.41	1.08 ± 0.39	1.10 ± 0.38	0.63
TC (mmol/l)	4.33 ± 1.24	4.43 ± 1.54	4.41 ± 1.04	4.38 ± 1.23	0.99
TG (mmol/l)	1.54 ± 0.67	1.59 ± 0.49	1.67 ± 0.61	1.64 ± 0.69	0.90
SBP(mmHg)	126.14 ± 5.90	128.04 ± 5.74	125.20 ± 6.46	127.50 ± 7.02	0.11
DBP(mmHg)	73.52 ± 6.82	71.92 ± 5.96	72.22 ± 6.26	74.52 ± 7.48	0.18
Cortical thickness (mm)	8.18 ± 0.58	9.22 ± 0.52	9.19 ± 0.32	8.89 ± 0.33*	< 0.01
Medullar thickness (mm)	9.53 ± 0.26	9.51 ± 0.26	9.57 ± 0.28	9.53 ± 0.25	0.76

Note:

*The cortical thickness values were significantly lower in the macro- group (*P* < 0.01).

BMI, body mass index; HbA1C, glycosylated hemoglobin; LDL, low-density lipoprotein; HDL, high-density lipoprotein; TC, cholesterol; TG, glycerin trilaurate; SBP, systolic pressure; DBP, diastolic pressure; control, control group; normo-, normal albuminuria; micro-, microalbuminuria; macro-, macroalbuminuria.

### Clinical characteristics

The weight and height of the participants were measured to calculate the body mass index (BMI). The blood pressure was measured at the right brachial artery in a seated position after a 15-min rest, and the mean value of three measurements was obtained. Venous blood, after an 8- to 12-h fast, was collected to measure HbA1C, fasting blood glucose, lipids, creatinine, urinary nitrogen, and albumin. All assays were performed according to routine procedures in the Biochemical Laboratory of the Affiliated Hospital of Qingdao University. The normal reference ranges for fasting blood glucose, HbA1C, and blood pressure were selected as less than 6.9 mM, 7.0%, and 130/80 mmHg, respectively. The urinary albumin excretion rate was presented as the ACR (mg of albumin/g of creatinine), which was confirmed with at least two separate urine samples. The evaluation of GFR was calculated by the Cockcroft-Gault formula, then verified by the Chinese People-improved MDRD formula for endogenous creatinine clearance (CCr) >30 ml/min, 1.73 m^2^). The Cockcroft-Gault formula used was CCr (ml/min) = (140-age) × weight (kg) / [72 × serum creatinine (mg/dL))] × (0.85 for female patients). The improved MDRD formula used was eGFR [ml/min, 1.73 m^2^)] = 186 × (Scr)^-1.154^ × (age)^-0.203^ × (0.742 for female patients).

### Imaging

All abdominal ultrasonography and SWV measurements were performed by a qualified radiologist with 15 years of relevant experience using a Siemens ACUSON S2000 US system (Siemens, Germany) equipped with a 3.5–5.5 MHz linear array transducer (4C1). The radiologists performed the examination had no knowledge of the clinical information of the study subjects. All subjects were initially screened by conventional ultrasonography for kidney evaluation. Patients were excluded if they had pathological conditions that may have affected measurements (such as cystic renal disease or parenchymal echogenicity change) or other abnormalities outside of the kidney but within the abdomen. If there were no pathological findings in the kidneys or other solid organs in the abdomen, then the subjects were included in the normal group. The cortex and medulla thicknesses were measured in the lower pole of the kidney (the perpendicular distance from the apex of the pyramid to the capsule of the kidney), and the average of the bilateral kidney was measured in the region of interest (ROI, box with fixed dimensions of 1 cm × 0.5 cm). An acoustic push pulse was transmitted immediately in the middle of the kidney cortex. Scanning was continued in the coronal section without pressure, and the scanning direction was constantly kept vertical with the kidney. Three valid measurements were obtained for each kidney at the same portion of the mid-pole, as parallel as possible to the radically arranged tubular system. The results were expressed as meters per second (m/sec), and the mean of three SWV values was used for the statistical analysis.

### Detection of BMP-7

A 3-ml sample of venous blood was obtained after the subjects had fasted for 8–12 h. After the sample was left standing for 30 min, it was centrifuged for 5 min, the serum was separated, and aliquots were stored at -70°C until use. The BMP-7 serum concentration were determined by enzyme-linked immunosorbent assay (ELISA) using BMP-7 (human) ELISA Kit (ELISA, Biovision, USA) according to the manufacturer’s instructions. One hundred μL serum was mixed with the assay buffer and incubated at room temperature for 45 min. The reaction OD was measured spectrometrically at 450 nm.

### Statistical analysis

SPSS version 17.0 software (IBM Corporation, Armonk, NY, USA) was used for all statistical analyses. Continuous variables were expressed as mean ± standard deviation; the χ^2^ test was used for categorical variables; and cortical thickness and clinical characteristics except for gender were analyzed by one-way analysis of variance and the least significant difference test for pair-wise comparisons, respectively. Differences were considered significant at *P* < 0.05. The ACR, BMP-7, and SWV were analyzed by the Kruskal-Wallis test and then the Mann-Whitney test for pairwise comparisons. Differences were considered to be significant at *P* < 0.01. Spearman analysis was used to determine the relationship between the SWV and ACR in DKD.

## Results

### Study participants and clinical characteristics

Through an initial screening, 326 subjects were excluded from the present study population, including 103 patients with nondiabetic renal diseases accompanying T2DM; 119 patients with a systemic disease/condition that could influence the albumin-creatinine ratio (ACR) as assessed by the medical history or examination; 79 patients taking medications that could affect renal function; and 25 patients with an anatomical distance between the body surface and kidney cortex greater than 5.0 cm.

Gender, age, fasting blood glucose, HbA1C, blood lipids, blood pressure, BMI, and medulla thickness of the patients are listed in Table 1; these parameters were not significantly different among the normo-, micro-, macroalbuminuria, and control groups (*P* > 0.05). However, the cortical thickness values were the lowest in the macroalbuminuria group among the three DKD groups (*P* < 0.01).

### BMP-7 levels in the DKD and control groups

**Fig 1:**
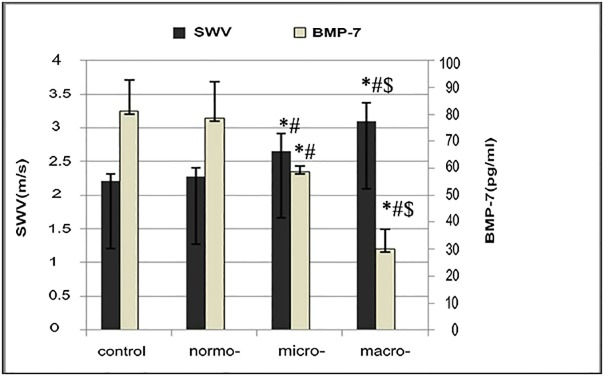
Comparison of SWV and BMP-7 between the groups. *Compared with the control group, *P* < 0.01; ^#^compared with the normo- group, *P* < 0.01; ^$^compared with the micro- group, *P* < 0.01. Both the micro- and macro- groups had a significantly higher SWV and lower BMP-7 compared to the normo- group (*P* < 0.01). SWV, shear wave velocity; BMP-7, bone morphogenetic protein-7; control, control group; normo-, normal albuminuria; micro-, microalbuminuria; macro-, macroalbuminuria.

Both the micro- and macro- groups had a significantly higher ACR compared to the normo- group (Table 2, *P* < 0.01). In addition, the serum BMP-7 level in the macro- group was significantly less than that in the micro- group (*P* < 0.01); and that in the micro- group was significantly less than that in the normo- group (*P* < 0.01). Moreover, the serum BMP-7 level in the micro- and macro- groups was significantly less than that in the control group (both *P* < 0.01); but there was no significant difference between the normo- and the control groups (*P* > 0.05) (see Table 2 and Fig. 1).

**Table 2:** SWV, ACR, and BMP-7 in the study groups(Median (Q1, Q3)).

Group	Cases	SWV (m/s)	BMP-7 (pg/ml)	ACR (mg of albumin/g of creatinine)
control	50	2.23 (2.14, 2.28)	82.18 (66.66, 96.51)	3.00 (2.95, 3.04)
normo-	50	2.31 (2.15, 2.39)	74.67 (71.61, 90.78)	8.00 (5.00, 12.00)
micro-	50	2.60 (2.45, 2.76)* ^#^	56.91 (53.71, 68.86)* ^#^	72.00 (51.25, 88.00)* ^#^
macro-	50	3.14 (2.95, 3.32)* ^#^ ^$^	25.82 (23.71, 37.78)* ^#^ ^$^	3211.00 (1060.00, 3466.25)* ^#^ ^$^

Note:

*Compared with the control group, *P* < 0.01;

^#^compared with the normo- group, *P* < 0.01;

^$^compared with the micro- group, *P* < 0.01.

Both the micro- and macro- groups had a significantly higher ACR, higher SWV and lower BMP-7 compared to the normo-group (*P* < 0.01).

SWV, shear wave velocity; BMP-7, bone morphogenetic protein-7; ACR, urinary albumin-creatinine ratio; control, control group; normo-, normal albuminuria; micro-, microalbuminuria; macro-, macroalbuminuria.

### SWV values in the DKD and the control groups

**Fig 2:**
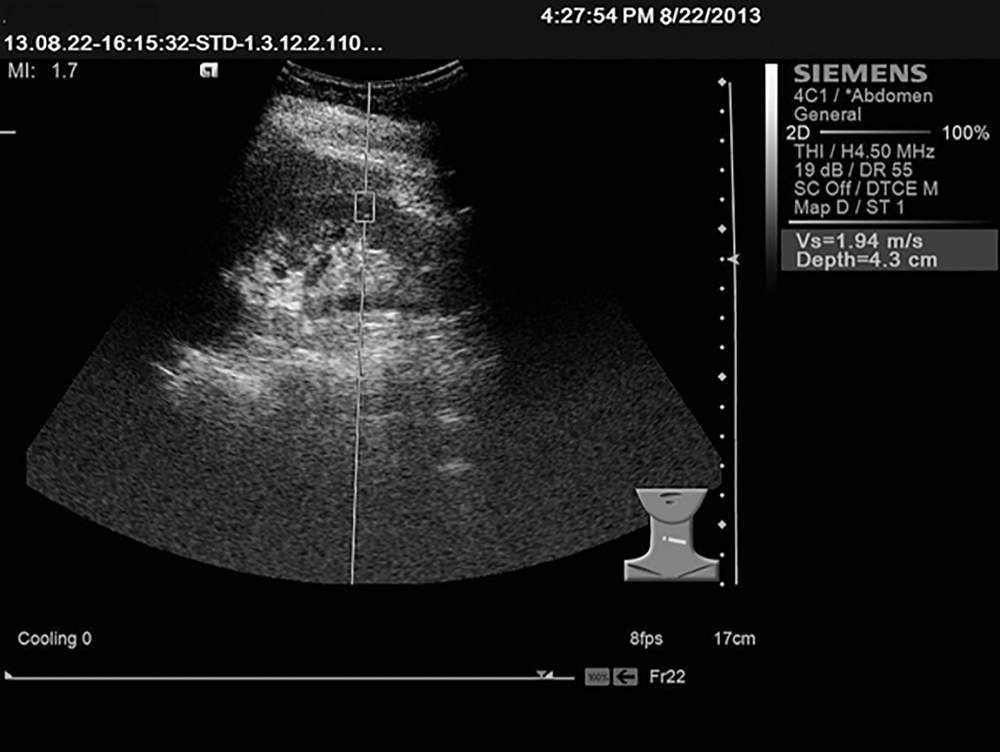
Shear wave velocity measurements. A Siemens ACUSON S2000 US system (Siemens, Germany) equipped with a 3.5–5.5 MHz linear array transducer (4C1) was used for the measurements. After measuring the cortex and medulla thicknesses in the lower pole of the kidney (the perpendicular distance from the apex of the pyramid to the capsule of the kidney), an acoustic push pulse was transmitted immediately in the region of interest (box with fixed dimensions of 1 cm × 0.5 cm). Three valid measurements were obtained for each kidney at the same portion of the mid-pole, as parallel as possible to the radically arranged tubular system. The results are expressed as meters per second (m/sec).

**Fig 3:**
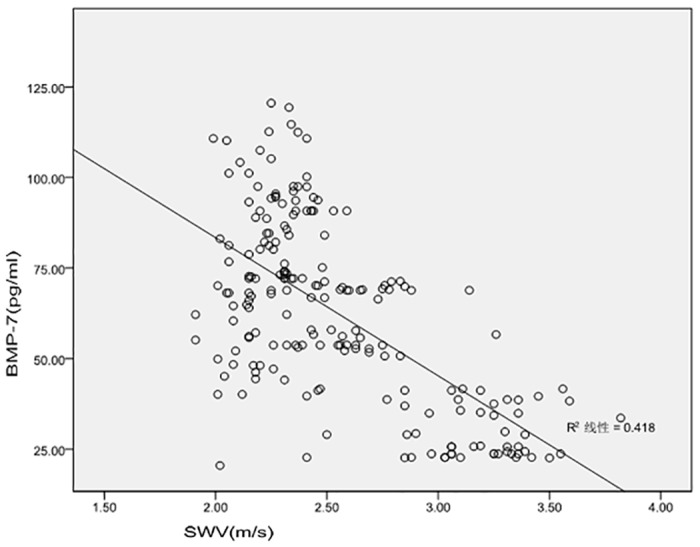
Correlation between SWV and BMP-7 in DKD subjects. SWV, shear wave velocity; BMP-7, bone morphogenetic protein-7; r = -0.569; *P* < 0.05; linear regression equation: y = -38.127x + 159.641.

The SWV values of all subjects were acquired successfully, and representative figure are depicted in Fig. 2 and summarized in Table 2. The mean SWV value of the macro- group was significantly greater than that of the micro- group, and that of the micro- group was significantly greater than that of the normo- group (*P* < 0.01). However, there was no significant difference between the normo- and the control groups (see Table 2 and Figs. 1 and 2). Next, we analyzed the correlation between the SWV and BMP-7 level; a significant and negative relationship was evident, with a correlation coefficient of-0.569 (*P* < 0.05, Fig. 3).

## Discussion

The present study showed that the SWV values increased and the BMP-7 levels decreased as the renal function decreased in patients with early DKD. In addition, there was a significant negative correlation between the two values (*P* < 0.05). We propose that a better clinical diagnosis of the degree of DKD is feasible by a combination of SWV and BMP-7 detections accompanied with the conventional diagnostic methods.

Our results were in agreement with previous discoveries demonstrating that lower levels of serum BMP-7 levels are associated with the progression of kidney disease [21] and kidney fibrosis [22]. The role of BMP-7 in renal pathophysiology also has been confirmed by an *in vivo* study, which showed that when DKD rats were given exogenous recombinant BMP-7, renal fibrosis and podocyte loss were attenuated[16,17]. In addition, Wong et al.[23] have shown that the participants who developed renal end points had significantly higher total TGF- β1 and lower BMP-7 levels, suggesting that TGF-β1 and BMP-7 are good predictors of renal progression in patients with T2DM. However, as BMP-7 levels may also change under other pathological conditions such as hepatic fibrosis[22], the results may be misinterpreted in patients suffering from these diseases, thereby interfering with the diagnostic specificity of the stage of kidney injury.

The elasticity of the kidney as determined by the SWV may be helpful in the early diagnosis of diabetic kidney damage[3]. As a noninvasive diagnostic method showing a direct reflection of renal stiffness, it is beneficial in clinical applications. In the present study, we demonstrated that the SWV values were significantly greater in the micro- and macro- groups than in the control group (*P* < 0.01) and that the differences between the normo- and micro- or macro- groups, and the micro- and the macro- groups were significant (all *P* < 0.01), suggesting that early DKD could cause significant renal pathological changes and lead to an increased in renal stiffness. With a significant negative correlation between the SWV and the ACR in the DKD groups, we suggest that an increased renal SWV could be related to the degree of renal dysfunction in early DKD. Therefore, renal SWV could be effectively used to diagnose early stages of DKD. The fact that no significant difference was observed in the SWV values between the normo- and the control groups may, at least partially, support that SWV values are relevant to the morphological condition, as no significant anatomical or chemical pathology change is expected between the normo- group and the control group. However, it has been reported that the SWV value can also be affected by renal blood flow. A lower SWV value was obtained in patients with a high brachial-ankle pulse wave velocity, an index of arterial stiffness[24]. As SWV and BMP-7 have their own advantages and disadvantages in the clinical diagnosis of DKD, they should be performed concurrently to minimize confounders in each methodology in patients with early DKD. However, a definite conclusion on such a benefit may still require further data analysis of patient results.

As there may be a discrepancy between ACR and eGFR in the assessment of renal function, especially at the early stage of the disease, albuminuria and symptomatic treatment have been the main clinical focus (up to end-stage kidney disease), so we selected ACR as an effective assessment parameter in our study on early DKD. However, a cohort study may be required to demonstrate that the combination of SWV and BMP-7 with ACR and/or eGFR as a confounder would be superior to that with ACR and/or eGFR alone in the diagnosis and assessment of the stage of early DKD. In addition, this study could not determine the relationship of the SWV and/or BMP-7, with the degree of renal damage in the absence of histological classification. However, a histopathological approach will be our next research priority to explore such a relationship.

In conclusion, we analyzed the SWV values and BMP-7 levels in patients with early DKD and demonstrated their usefulness in assessing the extent of renal damage. The SWV and serum BMP-7 level were correlated not only with each other, but also with ACR, and they significantly increased and decreased, respectively, as renal function declined. Combining the detection of SWV and serum BMP-7 level with conventional diagnostic methods may have a greater diagnostic value in the determination of the DKD stage than current methods.
